# Supplementary figures and images for: Identification of Toxoplasma calcium-dependent protein kinase 3 as a stress-activated elongation factor 2 kinase
Source: mSphere. 2023 Jun 5;8(4):e00156-23. doi: 10.1128/msphere.00156-23 (PMC10449493; doi:10.1128/msphere.00156-23)

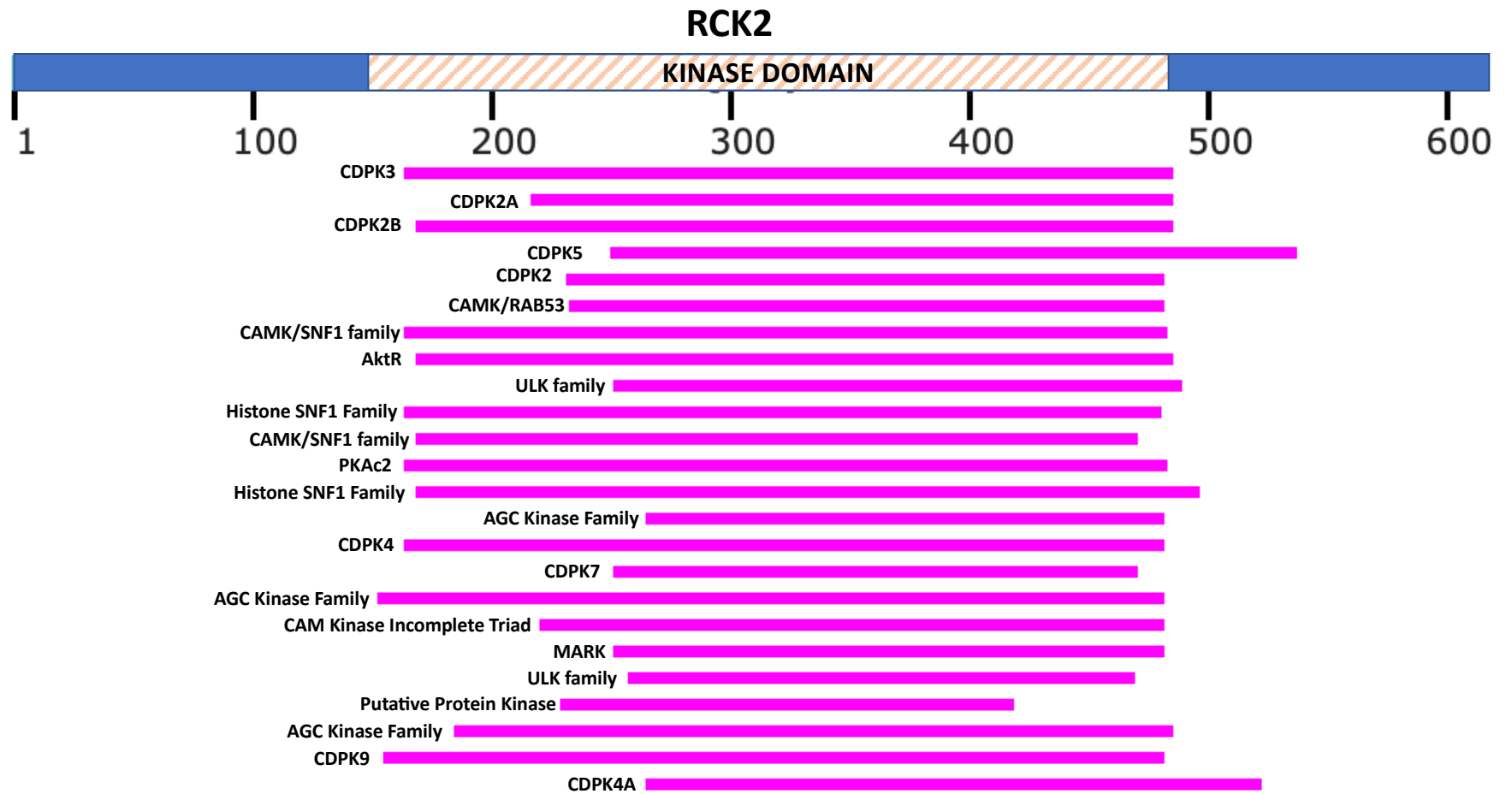

Supplement: Fig. S1 — Blast results. [file msphere.00156-23-s0001.pdf]
