# Supplementary material for: Identification of Toxoplasma calcium-dependent protein kinase 3 as a stress-activated elongation factor 2 kinase
Source: mSphere. 2023 Jun 5;8(4):e00156-23. doi: 10.1128/msphere.00156-23 (PMC10449493; doi:10.1128/msphere.00156-23)
Supplement: Supplemental File Legends/Descriptions — Legends for Fig. S1 and Table S1. [file msphere.00156-23-s0003.docx]

**SUPPLEMENT FILES LEGENDS/DESCRIPTIONS**

**Figure S1: Alignment of Top 24 Hits from Yeast RCK2 BLAST Analysis Against *Toxoplasma* Genome.** The yeast Rck2p protein sequences was used for BLAST analysis against the *Toxoplasma* ME49 reference genome using [www.toxoDB.org](http://www.toxoDB.org) Release 62. Shown are the top 25 hits as listed in the Table S1. Pink bars represent BLAST scores between 96-150. Rck2p drawing adapted from Reference #24.

**Table S1: Results of Yeast RCK2 BLAST Analysis Against *Toxoplasma* Genome.**

The yeast Rck2p protein sequences was used for BLAST analysis against the *Toxoplasma* ME49 reference genome using [www.toxoDB.org](http://www.toxoDB.org) Release 62. Spreadsheet contains the top 100 hits
